# Delays to anti-tuberculosis treatment intiation among cases on directly observed treatment short course in districts of southwestern Ethiopia: a cross sectional study

**DOI:** 10.1186/s12879-019-4089-x

**Published:** 2019-05-29

**Authors:** Abyot Asres, Degu Jerene, Wakgari Deressa

**Affiliations:** 1 Department of Public Health, College of Health Sciences, Mizan Tepi University, Mizan Aman, Ethiopia; 2 Management Science for Health, Addis Ababa, Ethiopia; 3 Department of Preventive Medicine, School of Public Health, College of Health Sciences, Addis Ababa University, Addis Ababa, Ethiopia

**Keywords:** Tuberculosis, Healthcare seeking, Patient delay, Provider delay, Total delay, Ethiopia

## Abstract

**Background:**

Delayed tuberculosis (TB) diagnosis and treatment increase morbidity, mortality, expenditure, and transmission in the community. This study assessed patient and provider related delays to diagnosis and treatment of TB.

**Methods:**

A cross-sectional study was conducted among 735 new adult TB cases registered between January to December 2015 in 10 *woredas* equivalent to districts of southwestern Ethiopia. Data were collected through face-to-face interview of patients within the first 2 months of treatment initiation. Delay in days was tracked at three intervals: between onset of symptoms and self-presentation (Patient delay), Self-presentation to treatment initiation (Provider delay) and total delay. Days elapsed beyond median were used to define the delays. Bivariate and multiple logistic regression models were fit to identify predictors of delays and statistical significance was judged at *p* < 0.05.

**Result:**

The median (inter-quartile range) of patient, provider and total delays were 25 (IQR;15–36), 22 (IQR:9–48) and 55 (IQR:32–100) days, respectively. More than half (54.6%) of the total delay was attributed to health system. Prior self-treatment [adjusted Odds Ratio (aOR)]: 1.72, 95% confidence interval [CI]:1.07–2.75), HIV co-infection (aOR:1.8, 95% CI: 1.05–3.10) and extra-pulmonary TB (aOR: 1.54,95% CI:1.03–2.29) were independently associated with increased odds of patient delay. On the other hand initial presentation to health posts or private clinics (aOR: 1.42, 95% CI: 1.01, 2.0) and patient delay (aOR: 1.81, 95% CI: 1.33–2.50) significantly predicted longer provider delay. Finally, having extra pulmonary TB (aOR: 1.6, 95% CI: 1.07–2.38), prior consultation of traditional healer (aOR: 3.72, 95% CI: 1.01–13.77) and use of holy water (aOR: 2.73, 95% CI: 1.11, 6.70) independently predicted longer total delay.

**Conclusion:**

Tuberculosis patients waited too long time to initiate anti-TB treatment reflecting longer periods of morbidity and disease transmission. The delays are attributed to the patient, disease and health system related factors. Hence, improving community awareness, involving informal providers, health extension workers and TB treatment supporters can reduce the patient delay. Similarly, cough screening and improving diagnostic efficiencies of healthcare facilities should be in place to reduce the provider delays.

**Electronic supplementary material:**

The online version of this article (10.1186/s12879-019-4089-x) contains supplementary material, which is available to authorized users.

## Background

Tuberculosis (TB) has been recognized as a global public health problem since 1993 when an estimated 7–8 million cases and 1.3–1.6 million deaths occured [1] . Since then, different global strategies have been designed and implemented for the control of TB; consequently an estimated 54 million lives were saved between 2000 and 2017 [2]. Despite such achievement, TB remained to be among the major global public health problems. Globally, 10 million incident cases and 1.3milion deaths were estimated to occur in 2017. Of the estimated incident cases, only 6.4 million (64%) were notified to National TB programs (NTPs) [2].

Tuberculosis is among the major public health problems in Ethiopia accounted for third cause of hospital admission and second cause of death [3]. Ethiopia is among the 30 TB High Burden Countries (HBC) where about 172,000 new cases and 25,000 deaths were estimated to occur in 2017. Out of the estimated cases in 2017, only 117,705 (68%) were notified to NTP [2]. The first national prevalence survey in 2011 revealed smear positive pulmonary TB (PTB) prevalence of 108/100,000. The survey further reported 55% of the cases were first identified by the survey and were not on treatment until the survey [4].

Early detection and treatment of TB cases have been a priority in the prevention and control of TB [5]. The delay to TB treatment can be categorized into: 1) patient delay that constitute time elapsed between onset of TB symptoms and first self presentation to formal care, 2) provider delay as time elapsed between first presentation to formal care and anti-TB treatment initiation and 3) total delay as time elapsed between onsets of TB symptoms and anti-TB treatment initiation. When diagnosis of TB is delayed, patients go without treatment for long and transmit the disease. In high prevalence settings, each infectious case of TB can result in up to 20 secondary infections until detection [6] . Moreover, delays to diagnosis and treatment of TB result in more serious illness by the time of diagnosis, increased length of infectiousness and poor treatment outcomes [7–11] and represent a time span for additional costs [12]. Consequently, delays to diagnosis has been emphasized as a major obstacle to TB control [13].

Systematic reviews of studies across the world reported median patient delay of 30 days and provider delay of 7-28 days [13, 14]. On the other hand, diagnostic delays ranging from a median of 8-191 days at different levels of health care delivery systems [7, 8, 15–18] were reported. Studies from different parts of Ethiopia also reported longer delays to initiate treatment for TB with median patient delay of 20–90 days [16, 17, 19] and provider delay of 6-34 days [17, 19–21]. A prevalence survey in northern Ethiopia [22] reported two-thirds of active TB cases were not detected timely. The long patient, provider and total delays are attributed to female gender, older age, low awareness about TB, repeated visits to health facilities, visiting lower level health care facilities and traditional healer [23, 24].

The studies in Ethiopia are limited to smear positive PTB cases [21], focused on cases presenting to either health centers [20] or hospitals [25] but not both. Moreover, the studies were conducted when TB had been treated for 8 months. Studies have shown that shorter treatment regimens could dramatically accelerate the reductions in TB incidence and mortality that can motivate suspects to seek care early and treatment [26]. In Ethiopia, treatment regimen of 6 months was introduced in 2011. Thus, complete picture of the patterns of delay of different categories of TB at different health care setups and in the era of reduced treatment regimen is scanty in the country. As a result the national TB research advisory council has set identification of barriers for the different forms of delays to TB treatment as a national priority research agenda [27]. However, recent evidences are lacking in Ethiopia in general and the study area in particular. Therefore, this study investigated time delays to initiate formal care seeking, diagnosis and treatment along with factors associated with the delays among new TB cases on treatment at health centers and hospitals in districts of southwestern Ethiopia.

## Methods

### Study design and setting

A cross-sectional study was conducted among newTB cases on treatment from January through December 2015 in three *zones* of Southern Nation Nationalities and Peoples Region (SNNPR); one of the nine Regional States in Ethiopia. The three study *Zones* include Bench Maji, Kaffa and Sheka together comprised of 26 *woredas* and four town administrations where about 2,064,102 peoples reside [28]. During the study, there were three hospitals and 65 health centers providing TB diagnosis and treatment based on national guidelines adopted from WHO [29, 30].

### Study population and sampling

The study population comprised of all new smear positive, smear negative and extra pulmonary TB cases older than 18 years and on anti-TB treatment at health centers and hospitals. Accordingly the sample size was computed using StatCalc program of EpiInfo using 95% significance level, 80% power, 38% expected proportion of illiteracy among those initiated formal healthcare seeking within 30 days of onset of illness and odds ratio (OR) 1.7 [31]. The calculation provided 486 cases, considering design effect of 1.5 and non-response of 10%, 802 new cases were required. Then ten woredas from the three zones were selected and all healthcare facilities providing TB diagnosis and treatment in the selected woredas were included for the study. Thus a total of 11 health centers and three hospitals were included. The samples were proportionally allocated to the zones, woredas and health facilities based on the TB caseloads reported during the preceding fiscal year to the study period. Finally, consecutive consenting new smear positive, smear negative and extra pulmonary cases within the first 2 months of anti-TB treatment and older than 18 years were enrolled until the required sample was reached.

### Data collection and analysis

Data were collected using structured questionnaire adapted from similar studies [4, 12, 32] and data abstraction checklist prepared from TB register and individual clinical chart. The questionnaire was translated into national language (*Amharic*) spoken by almost all residents in the study area. Ten diploma graduate nurse data collectors and three public health specialist (MPH) supervisors were recruited and trained for 3 days. Finally, new cases of TB were traced from the unit TB register and interviewed for their sociodemographic, health care seeking practices and knowledge towards TB.

Patient delay was assessed by asking the participants to recall or estimate the date or number of days elapsed between onset of TB constitutional symptoms (cough, fever, night sweats, chest pain, weight loss, loss of appetite) until they present to formal healthcare. Similarly, provider delay was estimated by asking date or number of days elapsed between first formal health care facility visit to final diagnosis and treatment initiation with anti-TB treatment. Finally, total delay was computed as a sum of patient and provider delay or number of days elapsed between onsets of illness to initiation of anti-TB treatment.

Knowledge about TB was assessed using eight items including cause of TB, TB is hereditary, TB is contagious, mode of TB transmission, (symptoms of TB, TB is curable, length of treatment and anti-TB drugs charge. Responses were recorded as 1 = yes as correct and 0 = no as wrong. The items internal consistency was checked (Cronbach’s Alpha (α) = 0.75) before computing an index. Finally, a knowledge index was computed from the scores and dichotomized in to good for those scored above median or poor otherwise.

Data were entered into Epi data, and processed on SPSS version 21. The data were described using frequency, proportions, measures of location and dispersion. Distribution numeric variables were assessed using normality plots (Q-Q plots and/or histograms) or Kolmogorov-Smirnov test for normality. The distribution of number of days elapsed across different time points were not normal and median days were used as a cutoff point to define delays. Thus patient, provider and total delays were defined based on median days elapsed.

Comparisons of medians across different groups was made using Manwhitney U and Kruskual Walis tests. Finally, bivariate and multiple binary logistic regression models were fitted to identify independent predictors of delays. Selection of the variables for multiple regression were made based on *p* value <=0.25 on crude analysis. The logistic model fitness was checked using Hosmer and Lemeshow test. In all the statistical tests, statistical significance was judged at *p* < 0.05.

Definition of terms

- **Patient delay:**days elapsed between onsets of illness to first formal healthcare seeking
- **Health system /provider delay:** is days spent between first consultation to initiation of treatments
- **Total delay:** number of days elapsed since onset of illness to anti-TB treatment initiation
- **Healthcare facility:** health institutions including health post, clinics, health center, and hospital organized to provide formal healthcare.
- **Care seeking:** consulting formal healthcare from healthcare facilities following illness.
- **New case of TB:** is a patient who never had treatment for TB, or has been on anti-TB treatment for less than 4 weeks in the past.

## Results

### Sociodemographic characteristics of the patients

A total of 735 TB cases from three hospitals and 11 health centers in the three *zones* were studied. Accordingly, 469(63.8%) and 266(37.2%) of the cases were registered at health centers and hospitals, respectively. The median age of the cases was 27 (inter-quartile range (IQR:20–37) years. Among the cases, 389(52.9%) and 216(29.4%) had completed elementary school and farmer, respectively (Table 1).

**Table 1:** Sociodemographic characteristics of TB cases on directly observed short course treatment (DOTS) in districts of southwestern Ethiopia, January to December 2015 (*n* = 735)

Variable		Frequency	Percent
Gender	Male	446	60.7
Age(years)	18–34	503	68.4
	35–65	216	29.4
	>65	16	2.2
Marital status	Never married	275	37.4
	Currently married	404	55.0
	Widowed/divorced	56	7.6
Educational status	No formal education	212	28.8
	Completed elementary	389	53.0
	Secondary and above	134)	18.2
Occupation	Employed	172	23.4
	Farming	216	29.4
	Unskilled work ^b^	51	6.9
	Dependants ^c^	296	40.3
Religion	Orthodox	300	40.8
	Muslim	104	14.1
	Catholic	4	0.5
	Protestant	314	42.7
	Traditional	13	1.8
Residence	Urban	369)	50.2
	Rural	366	49.8
Household size	Mean/SD	4.3/2.1	

^a^Standard deviation ^b^housemaid, daily laborer, ^c^ students, house wife, ^d^ father/ mother /husband/ wife/brother/sister/employer

### Knowledge towards tuberculosis

Almost all 721(98.1%) of the cases mentioned their illness as TB. Only 539(73.3%) had ever heard about TB before they were diagnosed as TB case. Among those ever heard about TB, 253(48.3%), 200(38.1%), 191(36.4%) and 190(35.3%) had heard from mass media, health facilities, TB patients and relatives respectively. The aggregated knowledge score about TB revealed, 545(74.1%) had scored above a median of 4.5 out of the eight items and labeled as having good knowledge.

### Delays to TB diagnosis and treatment

The median patient delay was 25 (IQR: 15–36) days. The median patient delay is significantly different across type of TB, educational status, marital status, and knowledge towards TB at *P* < 0.05(Additional file 1: Table S1). Three hundred seventeen (43.1%) cases perceived their first formal healthcare visit was delayed due to lack of money and waiting self limit was reported among 109 (26.1%) and 256 (80.8%), respectively. Clinically, cough, night sweating and fever were reported among 563(76.6%), 345(46.9%) and 300(40.8%) cases, respectively. While, 139(18.9%) of the cases took informal cares such as visiting traditional healer; only a third, 240(32.6%) consulted formal healthcare within 15 days of the onset of illness. The decision to ultimate visit to HCF was made upon referral and/or advice from relatives 280(38.6%), and TB patients on treatment 30(4.2%). Thus, 260 (35.4%) and 238(32.4%) of the cases first visited private clinics and public health centers respectively (Table 2).

**Table 2:** Initial symptoms encountered and Health care seeking pathways among new TB cases on directly observed treatment short course (DOTS), Southwestern Ethiopia, January to December 2015 (*n* = 735)

Variable		Frequency	Percent
Initial symptoms encountered	Cough	563	76.6
	Night sweat	345	46.9
	Fever	300	40.8
	Loss of appetite	279	38.0
	Chest pain	267	36.3
	Weight loss	236	32.1
	Haemoptysis	144	19.6
	Others^a^	62	8.4
First action to illness	Visit HCF	596	81.1
	Self-treatment	98	13.3
	Visit holy water	26	3.5
	Consult traditional healer	15	2.0
Perceived reason for not visiting HCF first (*n* = 149)	Thought illness limit by itself	97	65.1
	Perceived long waiting time at HCF	37	24.8
	Perceived expensive service fee	37	24.8
	HCF too far	29	19.5
	Other ^b^	22	14.8
First HCF visited	Private clinic	260	35.4
	Health center	238	32.4
	Hospital	221	30.1
	Health post	16	2.2
Source of advice/referral to visit first HCF	Self	472	64.2
	Parent/relative	280	38.1
	HEW ^c^	32	4.4
	TB patient	30	4.1
	HIV care clinic	16	2.2
	Other^d^	31	4.2
Travel time to first HCF visited	<=1 h	582	79.2
	> 1 h	153	20.8
Patient delay (days)	Median(IQR)	25(15–36)	
	Median (95%CI)	25(21,28)	

^a^neck swelling, head ache, joint pain, back pain, wound,^b^HCF closed, mistrust health care provider, bad previous experience at HCF, fear of HIV test and fear of TB diagnosis, ^c^HEW = Health Extension worker(trained females those provide household package of health care to household), ^d^drug shop(13), holy water(14), traditional healer(4)

Provider delay was at a median (IQR) of 22 (9–48) days. Among the included patients, 244(33.2%) and 112(15.2%) cases were diagnosed at the first visited HCF and during their first visit to HCF, respectively. Moreover, 623 (84.8%) cases were diagnosed after an average (SD) of 3.6 visits to an average (±SD) of 2.2 (1.2) HCFs (Table 3). Following the diagnosis of TB, 613(83.4%) of the cases were put on anti-TB treatment immediately and the rest after a median 2(range:1–7) days. The median provider delay is significantly correlated with patient delay (*r* = 0.2, *P* < 0.001) and different with type of TB and type of first visited HCF at *P* < 0.05 (Additional file 1: Table S1). Taken together, the median total delay was 55(IQR: 32–100) days to initiate anti-TB treatment. More than half (54.6%) of the total delay was attributed to provider and the rest to patient.

**Table 3:** Clinical characteristics and Pathways to initiate anti-TB treatment among cases on treatment Southwest Ethiopia, January to December 2015

Variable		Frequency (%)	Percent
Place TB diagnosis made	Hospital	448	61.0
	Health center	188	25.6
	Private clinic	99	13.5
Type of TB	Smear positive Pulmonary	373	50.3
	Smear negative Pulmonary	213	29.0
	Extra pulmonary	149	20.3
Mode of diagnosis	Bacteriological	373	50.7
	Clinical	362	49.3
Weight at treatment initiation	Mean(SD)Kg	48.8	
HIV status	Positive	68	9.3
	Negative	667	90.7
Receiving ART^a^ (*n* = 68)		27	39.7
Receiving CPT^b^ (*n* = 68)		32	47.1
Number of HCF visited ^c^	Median (IQR)	2(1–3)	
Number of visits made ^d^	Median (IQR)	3(2–4)	
Provider delay (days)	Median (IQR)	22(8–48)	
	Median(95%CI)	22(19,25)	
Total delay (days)	Median (IQR)	55(32–100)	
	Median (95%CI)	55(49,61)	

^a^Antiretroviral therapy ^b^ Cotrimoxazole Prohylactic therapy ^c^ number of HCF visited until diagnosis of TB is made, ^d^ number of total visits made to different HCF until TB diagnosis

### Factors associated with delays to anti-TB treatment

HIV co infection, having extra pulmonary TB, taking self-treatment before HCF visit, and traveling more than an hour to the first visited HCF increase the likelihood of patient delay beyond 25 days. On contrary, having good knowledge about TB decrease the odds of patient delay beyond 25 days.Those cases travelled more than an hour to reach the first visited HCF are 37% more likely to have patient delay beyondn25days. Having extra pulmonary TB and HIV co infection are 54 and 80% more likely to have patient delay beyond 25 days (Table 4).

**Table 4:** Factors associated with patient delay among TB patients on DOTS, southwestern Ethiopia January to December 2015

Variable		Patient delay(days)		COR (95% CI)	AOR (95%CI)
		>25 *n*(%)	<=25 *n*(%)		
Age				1.01(0.99,1.02)	1.01(1.001,1.03)*
Educational status	Illiterate	89(42.0)	123(58.0)	1.00	1.00
	Completed primary	215(55.3)	174(44.7)	1.05(0.75,1.47)	1.23(0.85,1.78)
	Secondary and above	63(47.0)	71(53.0	0.92(0.59,1.42)	1.27(0.77,2.1)
Type of TB	Pulmonary positive	175(47.9)	190(52.1)	1.00	1.00
	Pulmonary negative	104(47.7)	114(52.3)	0.95(0.68,1.32)	0.93(0.65,1.33)
	EPTB	91(59.9)	61(40.1)	1.51(1.03,2.21)	1.54(1.03,2.29)*
HIV status	Positive	41(60.3)	27(39.7)	1.52(0.92,2.52)	1.80(1.05,3.1)
	Negative	329(50.7)	338(49.3)	1.00	1.00
First action to illness	Self treatment	60(61.2)	38(38.8)	1.84(1.19,2.85)	1.72(1.07,2.75)*
	Traditional care	11(73.3)	4(26.7)	3.21(1.01,10.2)	2.98(0.91,9.72)
	Holy water	18(69.2)	8(30.8)	2.2(0.96,5.03)	2.01(0.86,4.67)
	Consult HCP	281	315	1.00	1.00
First visited HCF	Health post	13(81.3)	3(18.8)	1.00	1.00
	Health center	130(54.6)	108(45.4)	0.26(0.07,0.95)	0.25(0.07,0.94)*
	Hospital	98(44.3)	123(55.7)	0.18(0.0,0.64)	0.17(0.05,0.64)*
	Private clinic	129(49.6)	131(50.4)	0.22(0.06,0.79)	0.22(0.06,0.81)*
Travel time to first HCF	<=1 h	205(46.9)	232(53.1)	1.00	1.00
	>1 h	165(55.4)	133(44.6)	1.37(1.02,1.84)	1.37(1.01,1.88)*
Knowledge towards TB	Good	264(48.4)	281(51.6)	0.77(0.55,1.07)	0.67(0.46,0.98)*
	Poor	106(55.8)	84(44.2)	1.00	1.00

*statistically significant at *p* < 0.05

First presentation to non DOTS HCF, visiting more than one HCF until diagnoses, and having patient delay beyond 25 days more likely increase provider delay beyond 22 days. (Table 5). Those cases first visited non-DOTS center and having patient delay beyond 25 days are 42 and 81% more likely to have provider delay beyond 22 days (Table 5). Moreover, having extra pulmonary TB, first visitation to non DOTS HCF, consulting informal care before visiting formal care more likely to increase odds of total delay beyond 55 days (Additional file 1: Table S2).

**Table 5:** Factors associated with provider delay among TB patients on DOTS southwest Ethiopia, January to December 2015

Variable		Provider delay (days)		COR(95% CI)	AOR(95%CI)
		> 22 n(%)	<=22 n(%)		
Type of TB	Pulmonary positive	182(49.9)	183(50.1)	1.00	1.00
	Pulmonary negative	103(47.2)	115(52.8)	0.9(0.64,1.26)	0.87(0.60,1.25)
	Extra pulmonary	82(53.9)	70(46.1)	1.18(0.81,1.72)	1.08(0.72,1.62)
HIV status	Positive	39(57.4)	29(42.6)	1.38(0.84,2.30)	1.33(0.78,2.27)
	Negative	328(49.2)	339(50.8)	1.00	1.00
First action to illness	Self treatment	44 (44.9)	54(55.1)	0.81(0.53,1.25)	0.75(0.47,1.19)
	Traditional care	9(60.0)	6(40.0)	1.5(0.53,4.27)	1.19(0.47,3.51)
	Holy water	16(61.5)	10(38.5)	1.6(0.71,3.58)	1.28(0.55,2.99)
	Consult HCP	298(50.0)	298(50.0)	1.00	1.00
First visited HCF	DOTS center	203(44.2)	256(55.8)	1.00	1.00
	Non DOTS	164(59.4)	112(40.6)	1.85(1.36,2.50)	1.42(1.01,2.00)*
Number of visited HCF	1	233(62.0)	143(38.0)	2.77(2.05,3.73)	2.34(1.69,3.24)*
	> 1	133(37.2)	225(62.8)	1.00	1.00
Patient delay	Yes	213(57.6)	157(42.4)	1.80(1.36,2.50)	1.81(1.33,2.50)*
	No	154(42.2)	211(57.8)	1.00	1.00

HCP=Health Care Provider, *statistically significant at *p* < 0.05

## Discussion

Prompt detection and treatment of cases has been a priority in the prevention and control of TB that can be realized upon timely care seeking by patients and diagnosis by the health system. Our study assessed the delay and associated factors with the care seeking, diagnosis and treatment of TB in rural districts of southwestern Ethiopia. We found long patient, provider, and total delays, among TB cases on treatment. TB patients had waited for a median of 25 and 55 days respectively to initiate formal care seeking and anti-TB treatment. Both patients and provider delays contribute nearly equally to the total delay which is consistent with other studies from Ethiopia [20] and elsewhere [14]. The longer time elapsed since onset of illness to treatment initiation implies increased risk of morbidity and mortality among the cases and diseases transmission in the community [10, 33–35]. All forms of the delays are attributed to patient, disease and health system related factors. This implies the delays are multifaceted that calls for intensified TB case finding through strengthening healthcare facilities and health promotion activities.

Patients had waited for a median of 25 days until seeking help at formal health care providers which is consistent with 30 days in northern part of Ethiopia [17, 31, 36], 28 days in Uganda [14] and 30 days in Angola [13]. The relatively lower delay could be due to better access to HCF where nearly 80% of the cases traveled less than an hour to reach the first HCF. However, only a third (32%) of the cases had made visits to formal health care within 15 days of recommended timeline for TB suspects to visit HCF [29].Besides, more than half (57%) of the cases did not perceive their care seeking was delayed which implies the symptoms are taken as common and less severe to urge consultation of healthcare provider.

Before visits to formal health care facility, patients had taken variety of actions those influence timing of care seeking. Consistent with other studies, patients who took prior self-treatment were more likely to delay seeking formal care compared to those first consulted health care providers [37, 38]. This could be due to use of some home remedies or over the counter antibiotics or analgesics those might lessen the manifestation of the illness for the time being [39]. Moreover, those patients first visited traditional healer and holy water are more likely to have higher total delay. This could be due to the beliefs attached to the traditional care and holy water those might inhibit timely presentation and diagnosis [24].

Patients co infected with HIV are more likely to delay care seeking compared to those non- infected. This could be due to the alteration of classical clinical manifestations and signs of TB among HIV co infected patients [40]. On the other hand, patients suspected or tested HIV positive delay to present themselves to HCF due to fear of stigma attached to the co-occurrence of TB and HIV [9, 41, 42]. As a result, the high mortality among HIV co infected TB patients is partly explained by the delays to TB treatment [13, 43]. Those extra pulmonary cases patients are more likely to delay seeking care compared to the pulmonary cases. A similar finding was also reported form a study in northwest Ethiopia [25]. This could be due to the fact that extra pulmonary cases manifest with less severe and non-specific symptoms that urge patients to perceive the illness to be non-serious [44].

Patient’s perception and knowledge towards the TB disease and control activities do have impact on the care seeking practices. The study revealed that patients with good knowledge towards the TB disease and program are less likely to delay seeking care. Similarly, other studies have reported lack of knowledge and mistrust of the TB program as a reason for delayed care seeking [23, 45, 46]. In contrast, awareness and belief about TB’s curability is associated with longer patients delay [7, 14]. These imply need for awareness creation towards the TB illness and its control program to clear the paradox between the belief and longer delay.

The first consultation at HCF was made primarily (69.9%) at lower level public health care units (health posts and health centers) and private clinics. This is consistent with a study in Mediterranean countries that reported two thirds of patients first visited private sectors [46]. Nonetheless, diagnosis of TB was made primarily at hospital (61%) despite only few had made first visit to the hospitals. As a result, majority of the cases had made more than one visit to different HCFs until diagnosis. Similarly, studies from Uganda [14] and China [47] reported significant number of patients had visited more than one HCF until diagnosis. Subsequent to the missed opportunities during the repeated visits, diagnosis of TB had been made after a median of 22 days from the first visit to HCF. This is consistent with 21 days in a study from Amhara Region in Ethiopia [17] but higher than 6 in Addis Ababa [20] and 9 days in Tigray, northern Ethiopia [19]. The discrepancies could be due to the differences in accessibility to well equipped HCFs and skilled providers usually situated in cities like Addis Ababa. The longer provider delays due to the repeated visits portray high level of missed opportunities of early diagnosis that could have increased infectious period of the cases and costs incurred by the patients and households.

Longer provider delays had been reported among patients who first visited HCFs not providing DOTS services including health posts and private clinics. This is consistent with a study from Uzbekistan [38] and India [44].The higher provider delay at lower care units and private clinic could be due to lack of supplies, guidelines and skilled providers that enhance adherence to the national TB control program. In the current study, diagnosis of TB was mainly made at hospitals that depict unnecessary drugs and treatments provision at series of HCFs incurring extra cost. Besides the cost incurred, medications at the repeated visits themselves lead to delays to diagnosis and serious outcomes including drug resistance [48].On the other hand, the lower health care units and private clinics might lack proper logistics and skilled providers to timely diagnose the cases. Hence, expansions of the DOTS package to the lower and private clinic is required to curb the prevailing long delays.

Patients delayed to seek care are also more likely to have delayed diagnosis and treatment after they initiated care seeking. In contrast a study in Georgia [49] reported those patients with increased patient delay are less likely to have prolonged diagnostic delay. The discrepancy could be due to differences in measurement of the two delays. The higher odds of provider delay among those delayed to seek care can be explained by patients use of different forms of self-treatment and homemade remedies those might alter the manifestations of the TB illness that pose difficulties in timely diagnosis [39, 49]. Those patients delayed to seek care might have been ill for such long duration at which time productivity is compromised. Hence, the patients might be unable to cope with the costs required to have timely diagnosis even after initiating the care seeking. In addition, the long ill days are also associated with more severe disease at presentation [10] which hinders timely diagnosis [21].

Our study has few limitations. First, the study was carried out on those cases ultimately sought care and on treatment at the time of the study. Thus, measurement of delays relied on patient self-report that is liable to recall bias. We minimized this bias through interviewing patients soon after diagnosis and helping them to recall using local events. Second, the assessment of patients’ knowledge towards the TB diseases and its control program might be influenced by the information provided during treatment initiation. Therefore, this could have brought egg and chicken dilemma as the knowledge or care seeking preceded one to other. Third, we studied only new and adult cases so that the findings cannot be generalized to all forms of TB cases among all age groups. Fourth, we were not able to interview patients died during the intensive phase of treatment those could underestimated the true total delay attributed to the severe illness. Lastly our study lacked qualitative assessment of patients and health system those would have supplemented the quantitative data. On the other hand, consecutive enrollment of cases that minimized selection bias and triangulation of data sources from patient interview and chart reviews could be mentioned as strength of the study. Finally, our study is valid and generalizable to new adult cases in similar settings.

## Conclusions

Tuberculosis patients in the study area had passed through too long journey to initiate anti-TB treatment. The long journey is attributed to patient and provider delays those are positively correlated and contributed nearly equally to the total delay. The long delays at different levels of healthcare facilities portray high level of missed opportunities of early diagnosis of TB. The patient, provider, and total delays are attributed to the patient, disease and health system related attributes reflecting need for multifaceted intervention at all levels. Therefore, improving community awareness, involving informal providers, health extension workers and TB treatment supporters can reduce the patient delay. Similarly, cough screening and improving diagnostic efficiencies of healthcare facilities should be in place to reduce the provider delays.

## Additional file

Additional file 1: **Table S1.** Differences in median patient, provider and total delays among TB cases on DOTS, Southwestern Ethiopia, January to December 2015. **Table S2.** Factors associated with total delay among TB patients on DOTS, southwest Ethiopia January to December 2015 (DOCX 21 kb)

## Abbreviations

ART: Antiretroviral Therapy
CI: Confidence Interval
CPT: Cotrimoxazole Prophylactic Therapy
DOTS: Directly Observed Treatment Short course
EPTB: Extra pulmonary Tuberculosis
HBC: High Burden Countries
HCF: Healthcare Facility
HIV: Human Immunodeficiency Virus
IQR: Inter-quartile Range
OR: Odds Ratio
PTB: Pulmonary Tuberculosis
TB: Tuberculosis
WHO: World Health Organization
