# Supplementary material for: Delays to anti-tuberculosis treatment intiation among cases on directly observed treatment short course in districts of southwestern Ethiopia: a cross sectional study
Source: BMC Infect Dis. 2019 May 29;19:481. doi: 10.1186/s12879-019-4089-x (PMC6542087; doi:10.1186/s12879-019-4089-x)
Supplement: Supplementary file 1 — Table S1. Differences in median patient, provider and total delays among TB cases on DOTS, Southwestern Ethiopia, January to December 2015. Table S2. Factors associated with total delay among TB patients on DOTS, southwest Ethiopia January to December 2015 (DOCX 21 kb) [file 12879_2019_4089_MOESM1_ESM.docx]

Supplementary Table 1: Differences in median patient, provider and total delays among TB cases on DOTS, Southwestern Ethiopia, January to December 2015

| Variable | | Patient delay | | Provider delay | | Total delay | |
| --- | --- | --- | --- | --- | --- | --- | --- |
|  |  | Median | p value | Median | p value | Median | P value |
| Gender | Male | 25 | 0.9 | 22 | 0.9 | 54 | 0.7 |
|  | Female | 25 |  | 23 |  | 57 |  |
| Type of TB | Pulmonary positive | 23 | 0.03 | 22 | 0.02 | 53 | <0.001 |
|  | Pulmonary negative | 23 |  | 19 |  | 51 |  |
|  | Extra pulmonary | 29 |  | 26 |  | 67 |  |
| HIV result | Positive | 29 | 0.3 | 32 | 0.14 | 63 | 0.4 |
|  | Negative | 24 |  | 22 |  | 54 |  |
| Residence | Urban | 23 | 0.3 | 22 | 0.6 | 52 | 0.9 |
|  | Rural | 28 |  | 23 |  | 56 |  |
| Marital status | Single | 21 | 0.01 | 22 | 0.4 | 53 | 0.1 |
|  | Married | 27 |  | 22 |  | 55 |  |
|  | Divorced/widowed | 29 |  | 26 |  | 65 |  |
| Educational status | Illiterate | 26 | 0.04 | 18 | 0.03 | 48 | 0.14 |
|  | Primary | 26 |  | 26 |  | 59 |  |
|  | >=Secondary | 21 |  | 20 |  | 50 |  |
| First action taken | Visited HF | 22 | <0.001 | 23 | 0.4 | 53 | 0.001 |
|  | Other actions ^a^ | 30 |  | 22 |  | 64 |  |
| Travel time to nearby HF | <=1Hr | 21 | 0.001 | 22 | 0.9 | 53 | 0.2 |
|  | >1Hr | 29 |  | 23 |  | 58 |  |
| Knowledge on TB | Poor | 29 | 0.002 | 22 | 0.8 | 57 | 0.1 |
|  | Good | 22 |  | 22 |  | 54 |  |
| Occupation | Employed | 25 | 0.24 | 24 | 0.07 | 61 | 0.01 |
|  | Farming | 22 |  | 22 |  | 53 |  |
|  | Unskilled work | 31 |  | 35 |  | 91 |  |
|  | Dependents | 25 |  | 21 |  | 50 |  |
| First visited HCF | Health Post | 52 | 0.003 | 13 | <0.001 | 78 | 0.04 |
|  | Hospital | 22 |  | 17 |  | 42 |  |
|  | Health center | 29 |  | 19 |  | 55 |  |
|  | Private clinic | 23 |  | 30 |  | 62 |  |
| Visited >1HCF | Yes | 27 | 0.87 | 31 | <0.001 | 63 | <0.001 |
|  | NO | 23 |  | 14 |  | 41 |  |
| Place TB diagnosis made | HC | 30 | 0.002 | 18 | 0.005 | 54 | 0.3 |
|  | Hospital | 21 |  | 23 |  | 52 |  |
|  | Private clinic | 26 |  | 29 |  | 61 |  |

^a^self treatment, consult traditional healer, used holy water

Supplementary table 2 Factors associated with total delay among TB patients on DOTS, southwest Ethiopia January to December 2015

| Variable |  | Total delay | | Crude Odds ratio (COR) 95% CI | Adjusted Odds ratio (AOR) 95%CI |
| --- | --- | --- | --- | --- | --- |
|  |  | Yes | No |  |  |
| Residence | Urban | 180(48.9) | 188(51.1) | 1.00 | 1.00 |
|  | Rural | 185(50.4) | 182(49.6) | 1.06(0.80,1.42) | 1.26(0.90,1.77) |
| Educational status | Illiterate | 96(45.3) | 116(54.7) | 1.00 | 1.00 |
|  | Completed primary | 206(53.0) | 183(47.0) | 1.36(0.97,1.90) | 1.41(0.95,2.10) |
|  | Secondary and above | 63(47.0) | 71(53.0) | 1.07(0.69,1.65) | 1.27(0.74,2.17) |
| Type of TB | Pulmonary positive | 175(47.9) | 190(52.1) | 1.00 | 1.00 |
|  | Pulmonary negative | 101(46.3) | 117(53.7) | 0.94(0.67,1.31) | 0.95(0.67,1.36) |
|  | Extra pulmonary | 89(58.6) | 63(41.4) | 1.55(1.05,2.25) | **1.60(1.07,2.38)*** |
| HIV status | Positive | 38(55.9) | 30(44.1) | 1.32(0.80,2.18) | 1.36(0.79,2.35) |
|  | Negative | 327(49.0) | 340(51.0) | 1.00 | 1.00 |
| First action to illness | Self treatment | 49(50.0) | 49(50.0) | 1.09(0.71,1.67) | 0.91(0.58,1.43) |
|  | Traditional care | 12(80.0) | 3(20.0) | 4.36(1.22,15.63) | **3.72(1.01,13.77)*** |
|  | Holy water | 19(73.1) | 7(26.9) | 2.96(1.23,7.15) | **2.73(1.11,6.7)*** |
|  | Consult HCP | 285 | 311 | 1.00 | 1.00 |
| Travel time to nearby HCF | <=1hour | 210(48.1) | 227(51.9) | 1.00 | 1.00 |
|  | >1hour | 155(52.0) | 143(48.0) | 1.17(0.87,1.57) | 1.27(0.93,1.74) |
| Type of first visited HCF | DOTS center | 208(45.3) | 251(54.7) | 1.00 | 1.00 |
|  | Non DOTS | 157(56.9) | 119(43.1) | 1.59(1.18,2.15) | **1.63(1.19,2.24)*** |
| Knowledge towards TB | Good | 269(49.4) | 276(50.6) | 0.95(0.68,1.33) | 0.89(0.6,1.29) |
|  | Poor | 96(50.5) | 94(49.5) | 1 | 1 |
| Visited more than one HCF | Yes | 224(59.6) | 152(40.4) | 2.25(1.68,3.03) | **2.12(1.53,2.95)*** |
|  | No | 141(39.4) | 217(60.6) | 1.00 | 1.00 |
| Marital status | Married | 201(49.8) | 203(50.2) | 1.00 | 1.00 |
|  | Single | 131(47.6) | 144(52.4) | 0.92(0.67,1.25) | 0.80(0.55,1.15) |
|  | Divorced/widowed | 33(58.9) | 23(41.1) | 1.45(0.82,2.55) | 1.32(0.72,2.41) |
| Occupation | Employed | 89(51.7) | 83(48.3) | 1.00 | 1.00 |
|  | Farming | 103(47.7) | 113(52.3) | 0.85(0.57,1.27) | 0.83(0.51,1.34) |
|  | Unskilled work | 36(70.6) | 15(29.4) | 2.24(1.14,4.38) | **2.31(1.15,4.6)*** |
|  | Dependents | 137(46.3) | 159(53.7) | 0.80(0.55,1.17) | 0.87(0.57,1.33) |

*statistically significant at p<0.05
